# Molecular phylogeny of *Anopheles hyrcanus* group members based on ITS2 rDNA

**DOI:** 10.1186/s13071-017-2351-x

**Published:** 2017-09-07

**Authors:** Yuan Fang, Wen-Qi Shi, Yi Zhang

**Affiliations:** National Institute of Parasitic Diseases, Chinese Center for Disease Control and Prevention; WHO Collaborating Centre for Tropical Diseases; National Center for International Research on Tropical Diseases, Ministry of Science and Technology; Key Laboratory of Parasite and Vector Biology, Ministry of Health, Shanghai, 20025 People’s Republic of China

**Keywords:** *Anopheles*, Malaria, DNA barcoding

## Abstract

**Background:**

The *Anopheles hyrcanus* group includes 25 species, and is widely distributed in the Oriental and Palaearctic regions. Several species within this group are vectors of malaria, lymphatic filariasis and Japanese encephalitis. It is difficult or impossible to identify cryptic species based on their morphological characteristics, with some closely related species of the Hyrcanus Group have similar adult morphological characteristics. Thus, their molecular identification has been an important complementary method to traditional morphological taxonomy.

**Methods:**

We used 461 ribosomal DNA (rDNA) internal transcribed spacer 2 (ITS2) sequences relating to 19 species to reconstruct the molecular phylogeny of the Hyrcanus Group across its range. In addition, we compared the performance of rDNA ITS2 to that of mitochondrial DNA (mtDNA) cytochrome *c* oxidase subunit 1 gene (*cox*1) to assess the genetic divergence of Hyrcanus Group sibling species.

**Results:**

Based on Kimura’s 2-parameter (K2P) distance model, the average conspecific ITS2 divergence was 0.003, whereas sequence divergence between species averaged 0.480. Average ITS2 sequence divergences were almost 160 times higher among the Hyrcanus Group members than within each species. Two sets of sibling species, *An. lesteri* Baisas & Hu, 1936 and *An. paraliae* Sandosham, 1959; and *An. sinensis* Wiedemann, 1828, *An. belenrae* Rueda, 2005, and *An. kleini* Rueda, 2005, were resolved by ITS2. Each of these species was represented as an independent lineage in the phylogenetic tree. Results suggest that *An. pseudopictus* Grassi, 1899 and *An. hyrcanus* (Pallas, 1771) are most likely a single species. We uncovered two new ITS2 lineages that require further study before resolving their true taxonomic status, and designed a diagnostic polymerase chain reaction (PCR) assay to distinguish five morphologically similar species.

**Conclusions:**

Nuclear and mitochondrial genes generally provided consistent results for subgroup division. Compared to *cox*1, ITS2 is a more reliable tool for studying phylogenetic relationships among closely related mosquito taxa. Based on species-specific differences in ITS2 sequences, the multiplex PCR assay developed here can be used to improve the efficiency of vector identification. Thus, this research will promote the progress of malaria vector surveillance in both epidemic and non-epidemic areas of South and East Asia.

**Electronic supplementary material:**

The online version of this article (10.1186/s13071-017-2351-x) contains supplementary material, which is available to authorized users.

## Background

The *Anopheles hyrcanus* group (hereinafter Hyrcanus Group) includes (at least) 25 species and belongs to the *Anopheles* Myzorhynchus Series, with one provisionally designated member [1, 2]. The group is widely distributed in Oriental and Palaearctic regions. *Anopheles sinensis* and *An. lesteri* are the main malaria vectors in China [3], *An. hyrcanus* is a potential malaria vector in southern France [4, 5], *An. kleini* and *An. pullus* Yamada, 1937 are primary vectors of malaria in the Republic of Korea [6], and *An. sinensis*, *An. nigerrimus* Giles, 1900, and *An. peditaeniatus* (Leicester, 1908) are potential vectors of malaria in Thailand [7, 8].

Because many of the primary malaria vectors belong to the Hyrcanus Group, accurate species identification and phylogenetic relationship evaluation in this group are essential for understanding malaria transmission and its relationship with the evolution of *Plasmodium* spp. However, species within the Hyrcanus Group are difficult or impossible to be accurately distinguished using only morphological characteristics, even for trained taxonomists [9, 10], due to highly variable morphologies and to adults of some species possessing almost identical adult morphological features, as is the case of the most closely related species [11, 12].

DNA barcoding is an important addition to traditional morphology-based methods and a highly useful tool for species recognition, regardless of life stage [13–15]. The mitochondrial cytochrome *c* oxidase subunit 1 gene (*cox*1) is the standard barcode for species identification in a wide range of animal taxa, and is approximately 658 bp long [16, 17]. However, genomic introgression frequently involves mitochondrial DNA (mtDNA), because of recent hybridization events between species [18, 19]. Recent hybridization transfers mtDNA from one species to another and can lead to mtDNA variation [18, 20, 21]. In our previous study [22], *cox*1 failed to distinguish between recently diverged taxa, suggesting that mtDNA may not be appropriate for investigating the molecular phylogeny of the Hyrcanus Group.

A DNA marker that evolves at a species-level rate would be able to accurately reconstruct phylogenetic relationships within the Hyrcanus Group, and elucidate the ambiguity that has arisen from improper classification [23, 24]. Internal transcribed spacer 2 (ITS2) has been used for addressing taxonomic issues within the Hyrcanus Group because it has high interspecific, and low intraspecific variability [11, 12, 16, 25–27]. Using this marker, three newly proposed lineages have been revealed: two separated from *An. sinensis*, namely *An. belenrae* and *An. kleini* [28], and one closely related to *An. hyrcanus*, provisionally designated as *An. hyrcanus* sp_IR_ [16]. In addition, based on interspecific comparisons of ITS2, *An. yatsushiroensis* was synonymized with *An. pullus* [29, 30], *An. kunmingensis* and *An. liangshanensis* Kang, Tan, Cao, Cheng, Yang & Huang, 1984 were found to be synonymous [31, 32], *An. paraliae* was considered a synonym of *An. lesteri* [33], *An. anthropophagus* and *An. lesteri* were classified as conspecific [34, 35], and *An. kleini* was presumed a synonym of *An. engarensis* Kanda & Oguma, 1978 [12].

To reconstruct the molecular phylogeny of the Hyrcanus Group, the barcoding gap of ITS2 should be identified. Therefore, it is necessary to include specimens of the same species collected from different geographical locations [17, 36], in order to calculate the intra- and interspecific variation of ITS2 within the group. The ITS2 sequence database in GenBank (http://www.ncbi.nlm.nih.gov/genbank) permits the use of reference sequences for Hyrcanus Group species identification across a relatively wide geographical distribution [33]. Thus, in the present study, GenBank sequences and our original data were used to reconstruct a phylogeny for the Hyrcanus Group based on ITS2, to resolve phylogenetic relationships between closely related taxa. In addition, we compared rDNA ITS2 and mtDNA *cox*1 in terms of genetic divergence and distinction efficiency among Hyrcanus Group species. Using conserved ITS2 fragments from Hyrcanus Group species, we also designed a diagnostic polymerase chain reaction (PCR) assay for distinguishing five morphologically similar species. The present study will help improve the efficiency of mosquito molecular identification in malaria vector surveillance.

## Methods

### ITS2 sequence retrieval from GenBank

We used the keywords “(species name) & ITS2” to search for ITS2 sequences of Hyrcanus Group members deposited in GenBank. Details of these sequences are provided in Additional file 1: Table S1.

### DNA extraction, ITS2 amplification and sequencing

Dry, pin-mounted museum specimens of the Hyrcanus Group collected after the year 2000, and identified using standard taxonomic keys [37, 38], were used for DNA extraction following the protocol specified in a previous paper [22]. One leg was removed from each adult specimen, transferred to a dry 1.5 ml Eppendorf tube, ground to powder, and incubated in lysis buffer overnight at 56 °C. Bind, wash, and elution steps followed the manufacturer’s instructions for the Qiagen DNA Blood and Tissue Kit (Qiagen, Hilden, Germany). Voucher specimens were stored in the Herbarium of the National Institute of Parasitic Diseases, Chinese Center for Disease Control and Prevention (NIPD, China CDC).

Approximately 550 bp PCR products were amplified using the forward primer ITS2*a* (5′-TGT GAA CTG CAG GAC ACA T-3′) and the reverse primer ITS2*b* (5′-TAT GCT TAA ATT CAG GGG GT-3′) [39]. The 25 μl reaction mixture contained 12.5 μl of 2× *Taq* PCR Master Mix with dyes (DBI Bioscience, Shanghai, China), 1 μl of each 10 μM primer, 4 μl of extracted DNA, and 6.5 μl of ddH_2_O. The thermocycling profile consisted of an initial denaturation at 95 °C for 2 min followed by 35 cycles at 95 °C for 1 min, 55 °C for 1 min, and 72 °C for 2 min, with a final extension at 72 °C for 7 min. Amplicons were subjected to electrophoresis on a 1.2% agarose gel stained with GoldView dye (Solarbio, Beijing, China), and cleaned and sequenced by Sangon (Shanghai, China).

### Sequence annotation, alignment, and phylogenetic analysis

Our original ITS2 sequences, and those deposited in GenBank, were annotated using the ITS2 annotation tool (http://its2.bioapps.biozentrum.uni-wuerzburg.de) [40]. A multiple sequence alignment was conducted in ClustalW2 [41] and manually adjusted where necessary. Gaps were excluded from the analysis and characters were unweighted. *Anopheles lindesayi* Giles, 1900 (AJ620898) and *An. claviger* (Meigen, 1804) (AY129232 and DQ229313) were used as outgroup taxa to the Hyrcanus Group, following previous studies [42]. The neighbour-joining (NJ) method generally reveals shallow intraspecific and deep interspecific divergences [17, 43]; thus, a bootstrapped NJ tree was constructed using 1000 replicates by ClustalW2 to provide a graphical representation of the phylogenetic relationships among Hyrcanus Group members. This phylogram was visualized using Figtree v1.4.2 [44].

### Genetic diversity analysis and neutrality test

Both intraspecific and interspecific ITS2 divergences were examined using Kimura’s 2-parameter (K2P) distance model [45] in MEGA v5.1 [46]. Genetic divergence, according to Nei’s distance model [47], was determined in Arlequin v3.5.2.2 [48]. Genetic diversity indices and neutrality tests (Fu’s *Fs* [49] and Tajima’s *D* [50]) were calculated using DnaSP v5.10 [51].

### Primer design and conditions of the multiplex PCR assay

We developed a multiplex PCR-based assay to simultaneously identify five Hyrcanus Group species, *An. sinensis*, *An. lesteri*, *An. peditaeniatus*, *An. hycanus* and *An. pullus*, most of which are sympatric. Species-specific reverse primers were designed using Primer3web (http://primer3.ut.ee/), based on the distinctive set of ITS2 sequences of each species. Forward primers applied the universal primer ITS2*a*, which is located on the conserved 5.8S gene. The species-specific reverse primers were as follows: *An. peditaeniatus*, 5′-ACG GCG TAG GTT ATT GTC TCT-3′; *An. hyrcanus*, 5′-GGY TTT ACA CCG CAG TTC TT-3′; *An. lesteri*, 5′-GCC CAT TCC MCT ATC TCG AA-3′; *An. pullus*, 5′-CGC TCT CTC AAC AAC TGG GT-3′; and *An. sinensis*, 5′-GAG TGG CCT CAC TCT TGG AG-3′. The multiplex PCR was conducted in 25 μl total volume, containing 12.5 μl of 2× *Taq* PCR Master Mix (with dyes), 1.5 μl of 10 μM ITS2*a*, 0.75 μl of each 10 μM species-specific reverse primer, 2 μl of extracted DNA, and 5.25 μl of ddH_2_O. Conditions for the amplification comprised an initial denaturation at 95 °C for 2 min followed by 35 cycles at 95 °C for 30 s, 58 °C for 30 s, and 72 °C for 30 s, with a final extension at 72 °C for 5 min. The amplicons were separated by electrophoresis on 1.2% agarose gels in 0.5× Tris-borate-EDTA (TBE) buffer and stained with GoldView dye (Solarbio, Beijing, China).

### Evaluation of the multiplex PCR assay

*Anopheles liangshanensis*, *An. jeyporiensis* James, 1902, *An. minimus* Theobald, 1901, *An. harrisoni* Harbach & Manguin, 2007, *An. maculatus* Theobald, 1901, *An. aconitus* Dönitz, 1902, *An. splendidus* Koidzumi, 1920 and *An. dravidicus* Christophers, 1924 are common in China. The DNA of several *Anopheles* species (see Additional file 2: Table S2) previously identified by molecular methods in our lab was used to validate the novel multiplex PCR assay. To determine the sensitivity of the assay, it was further tested using a DNA dilution series for each of the five target species from 10 ng/μl to 1 × 10^-5^ ng/μl or 1 × 10^-6^ ng/μl.

## Results

### Sequence analysis

Four hundred and forty one ITS2 sequences of the Hyrcanus Group were extracted from GenBank. Details of the ITS2 sequences used for the phylogenetic analysis are given in Additional file 1: Table S1. Seven ITS2 sequences were distant from conspecific sequences, and therefore were excluded from further phylogenetic analyses. We could not find identical sequences in GenBank for two of these sequences. One questionable sequence for *An. crawfordi* Reid, 1953 (AF261949) was distant from conspecific individuals; its closest sequences were from *An. peditaeniatus*, with a similarity of 83–84%. In our previous study [22], two peculiar *cox*1 sequences for *An. crawfordi* (KF830735 and KF830736) clustered with sequences for *An. xui* Dong, Zhou, Dong & Mao, 2007. Unfortunately, there is no current ITS2 record of *An. xui*. The sequence AF261949 might have been obtained from an *An. xui* specimen, but more rigorous morphological and genetic examinations are needed to confirm this. The other sequence for *An. hyrcanus* was detected as accession number KC769647, and its closest sequences were from *An. sineroides* Yamada, 1924 (90% similarity). The original specimen was from China; however, no detailed information about the collection site was available. We obtained four ITS2 sequences of *An. hyrcanus* from Xinjiang province, the only region of China in which it is found [37]; therefore, specimen KC769647 was probably collected in Xinjiang Province as well. It is unlikely that specimens from the same province would have such large genetic distances between them, so it is possible that the specimen KC769647 belongs to a cryptic species, or to another Hyrcanus Group member that has no available ITS2 data.

Five *An. sinensis* individuals (KJ462254–KJ462258) were much closer to *An. crawfordi* individuals (99% similarity with AB779132) than to some of their conspecifics, and clustered with the *An. crawfordi* lineage in the phylogenetic tree. Thus, these sequences might be incorrect, presumably due to the misidentification of original specimens.

**Fig. 1:**
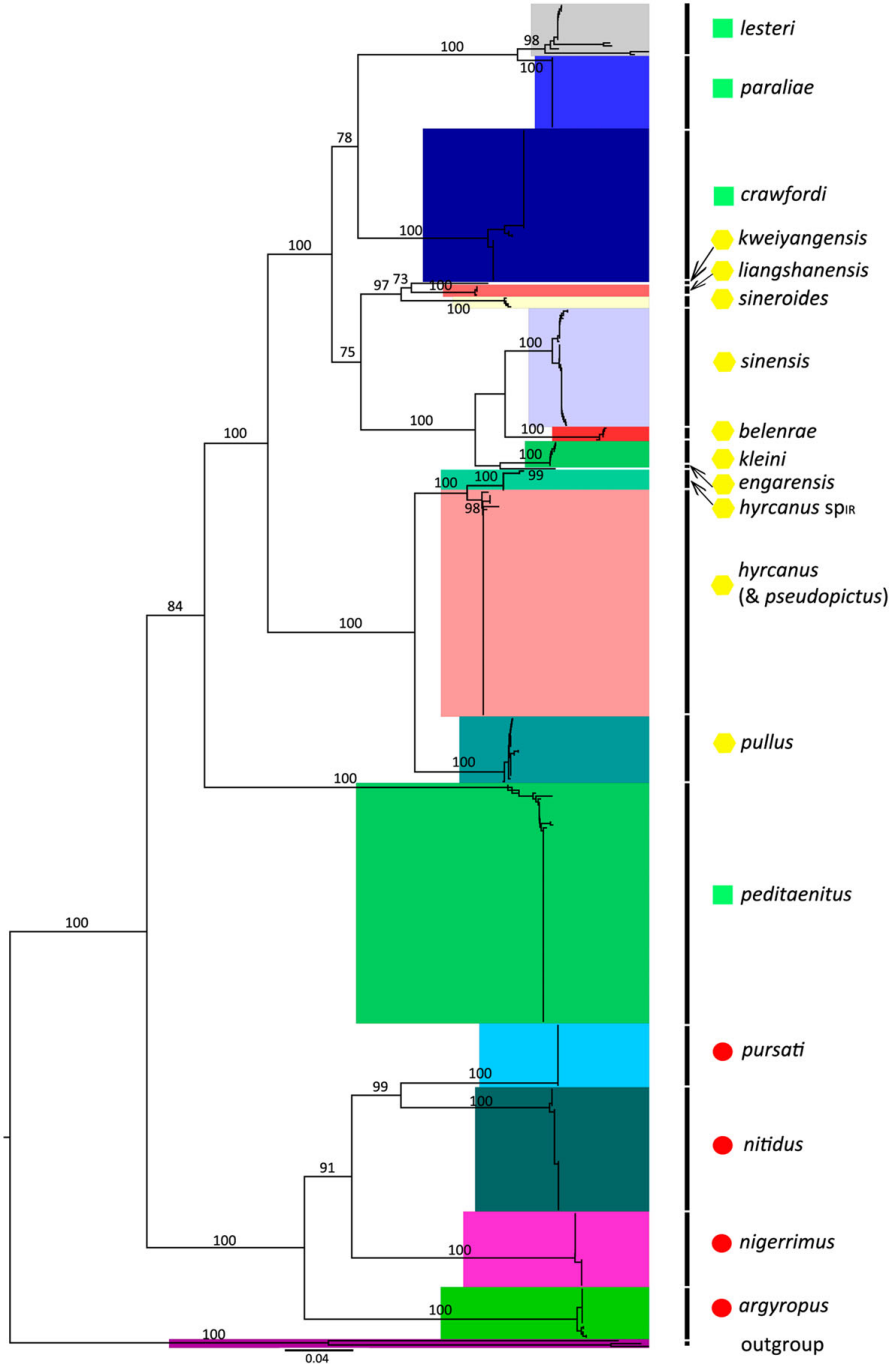
Neighbour-joining phylogenetic tree of the Hyrcanus Group based on ITS2 sequences from GenBank and our original data. Bootstrap values (1000 replicates) of neighbour-joining analyses are shown above/below the main lineages. Lineage designation is indicated on the right. The geometric shapes correspond to different subgroups of the Hyrcanus Group, according to the classification of Harbach [1], Lesteri Subgroup; Nigerrimus Subgroup; unclassified species. Bars represent 0.04 substitutions per site. *Anopheles lindesayi* and *An. claviger* were used as the outgroup taxa

The ITS2 sequences KJ960222–KJ960226, collected from Iran, were originally identified as *An. hyrcanus*. In the alignment of the Hyrcanus Group members, their nucleotide substitutions were more similar to those of *An. hyrcanus* sp_IR_ sequences than to *An. hyrcanus* sequences, particularly when considering that all had an 11 bp insertion [16]. In the NJ tree (Fig. 1), they clustered with the *An. hyrcanus* sp_IR_ lineage and were separated from *An. hyrcanus*, suggesting they might be sequences of *An. hyrcanus* sp_IR_.

Twenty-seven ITS2 sequences belonging to *An. sinensis*, *An. peditaeniatus*, *An. pullus*, *An. hyrcanus*, *An. lesteri*, and *An. liangshanensis* were generated during this study and have been submitted to GenBank; their accession numbers and collection sites are presented in Table 1.

**Table 1:** List of ITS2 sequences obtained from this study

Species	Geographical localities	GenBank ID
*An. sinensis*	China: Yunnan Prov.	KU312198
	China: Yunnan Prov.	KU312199
	China: Yunnan Prov.	KU312200
	China: Yunnan Prov.	KU312201
	China: Yunnan Prov.	KU312202
	China: Yunnan Prov.	KU312203
*An. peditaeniatus*	China: Yunnan Prov.	KU312204
	China: Yunnan Prov.	KU312205
	China: Yunnan Prov.	KU312206
	China: Yunnan Prov.	KU312207
	China: Tibet	KU312208
	China: Tibet	KU312209
*An. pullus*	China: Shangdong Prov.	KU312210
	China: Liaoning Prov.	KU312211
	China: Liaoning Prov.	KU312212
	China: Liaoning Prov.	KU312213
	China: Liaoning Prov.	KU312214
	China: Liaoning Prov.	KU312215
	China: Shangdong Prov.	KU312216
*An. hyrcanus*	China: Xinjiang Prov.	KU312217
	China: Xinjiang Prov.	KU312218
	China: Xinjiang Prov.	KU312219
	China: Xinjiang Prov.	KU312220
*An. lesteri*	China: Liaoning Prov.	KU312221
	China: Liaoning Prov.	KU682193
*An. liangshanensis*	China: Yunnan Prov.	KU682194
	China: Yunnan Prov.	KU682195

### Intra- and interspecific ITS2 variation

**Fig. 2:**
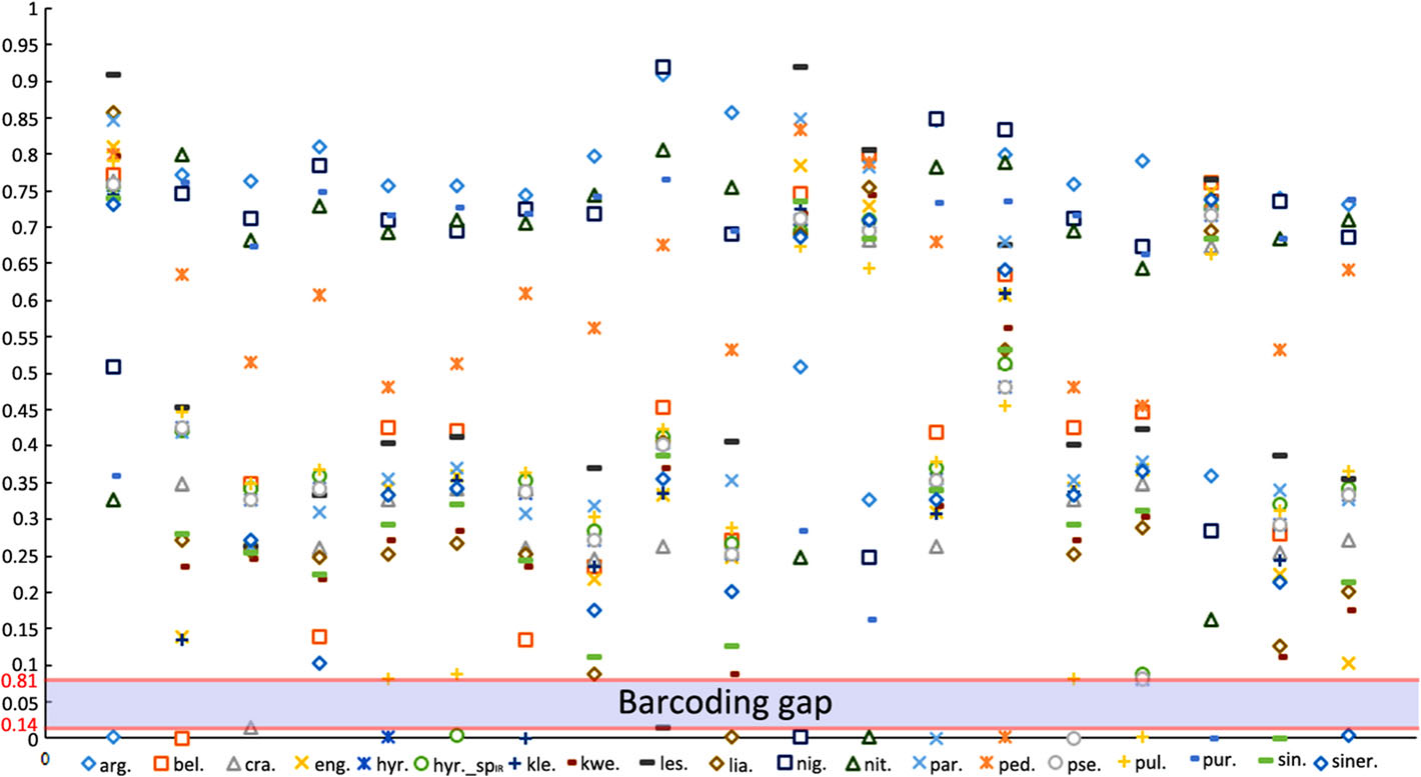
Intra- and interspecific ITS2 divergences in 19 Hyrcanus Group members determined using Kimura’s 2-parameter distance. The barcoding gap ranged 0.014–0.081. Y-axis, genetic divergence; x-axis, Hyrcanus Group members

Individual species were represented by one to 83 individuals, for a total of 461 ITS2 sequences (Table 2). Almost all species possessed a distinctive set of ITS2 sequences. The intra- and interspecific divergences of ITS2 in the Hyrcanus Group are shown in Table 2, and Fig. 2 shows the distribution of pairwise K2P genetic distances between ITS2 sequences, revealing a distinct barcoding gap. The average intraspecific K2P distance was 0.003. No intraspecific variation was found for *An. belenrae*, *An. kleini*, *An. paraliae* or *An. pursati* Laveran, 1902. Deep divergences were detected in two species, *An. lesteri* (0.0142) and *An. crawfordi* (0.0143). Short distances were observed between some pairs of species: *An. hyrcanus* and *An. pseudopictus* (0.001), *An. hyrcanus* sp_IR_ and *An. pseudopictus* (0.020), *An. hyrcanus* and *An. hyrcanus* sp_IR_ (0.021), *An. lesteri* and *An. paraliae* (0.048), and *An. kleini* and *An. engarensis* (0.072). This was confirmed by the Nei’s genetic distance obtained (Additional file 3: Figure S1). The taxonomic validity of *An. pseudopictus*, *An. hyrcanus* sp_IR_, *An. paraliae*, and *An. kleini* is still controversial [1, 4, 12, 16, 33], and further studies are needed to resolve these uncertainties; we excluded the above data in the calculation of the mean interspecific distances. Therefore, interspecific K2P distances ranged from 0.081 between *An. pullus* and *An. pseudopictus* to 0.920 between *An. nigerrimus* and *An. lesteri*, with an average of 0.480. Hence, the ITS2 sequence divergences among intragroup species were, on average, almost 160 times higher than the average divergences within species. The ITS2 barcoding gap was 0.014–0.081 (Fig. 2), suggesting that the ITS2 spacer is a good marker for differentiating Hyrcanus Group members.

**Table 2:** Mean intra- and interspecific K2P distances of the ITS2 sequence in 19 Hyrcanus Group members

Species	*n*	arg.	bel.	cra.	eng.	hyr.	hyr. sp_IR_	kle.	kwe.	les.	lia.	nig.	nit.	par.	ped.	pse.	pul.	pur.	siner.	sin.
arg.	18	**0.001**	–	–	–	–	–	–	–	–	–	–	–	–	–	–	–	–	–	–
bel.	5	0.772	**0.000**	–	–	–	–	–	–	–	–	–	–	–	–	–	–	–	–	–
cra.	53	0.763	0.348	**0.014** ^**b**^	–	–	–	–	–	–	–	–	–	–	–	–	–	–	–	–
eng.	1	0.810	0.138	0.261	na	–	–	–	–	–	–	–	–	–	–	–	–	–	–	–
hyr.	48	0.757	0.425	0.326	0.341	**0.001**	–	–	–	–	–	–	–	–	–	–	–	–	–	–
hyr. sp_IR_	7	0.758	0.421	0.342	0.358	0.021^a^	**0.003**	–	–	–	–	–	–	–	–	–	–	–	–	–
kle.	9	0.744	0.134	0.262	0.072^a^	0.336	0.352	**0.000**	–	–	–	–	–	–	–	–	–	–	–	–
kwe.	1	0.797	0.236	0.245	0.218	0.271	0.285	0.236	na	–	–	–	–	–	–	–	–	–	–	–
les.	18	0.910	0.453	0.264	0.334	0.404	0.412	0.336	0.370	**0.014** ^**b**^	–	–	–	–	–	–	–	–	–	–
lia.	4	0.858	0.270	0.257	0.247	0.253	0.266	0.253	0.088	0.405	**0.002**	–	–	–	–	–	–	–	–	–
nig.	26	0.508	0.746	0.711	0.784	0.710	0.694	0.725	0.719	0.920	0.691	**0.002**	–	–	–	–	–	–	–	–
nit.	43	0.328	0.800	0.681	0.729	0.693	0.710	0.706	0.743	0.806	0.754	0.248	**0.002**	–	–	–	–	–	–	–
par.	25	0.848	0.418	0.264	0.310	0.355	0.370	0.307	0.318	0.048^a^	0.353	0.849	0.784	**0.000**	–	–	–	–	–	–
ped.	83	0.800	0.635	0.515	0.608	0.481	0.514	0.610	0.562	0.675	0.533	0.833	0.789	0.681	**0.001**	–	–	–	–	–
pse.	30	0.760	0.426	0.327	0.342	0.001^a^	0.020^a^	0.337	0.271	0.403	0.253	0.712	0.695	0.353	0.482	**0.000**	–	–	–	–
pul.	23	0.792	0.446	0.349	0.368	0.082	0.087	0.363	0.303	0.423	0.290	0.674	0.644	0.379	0.456	0.081^b^	**0.001**	–	–	–
pur.	22	0.359	0.760	0.674	0.748	0.716	0.728	0.719	0.742	0.765	0.694	0.284	0.161	0.732	0.736	0.716	0.663	**0.000**	–	–
siner.	4	0.739	0.280	0.254	0.225	0.293	0.322	0.243	0.111	0.388	0.126	0.736	0.684	0.340	0.531	0.293	0.312	0.685	**0.000**	–
sin.	41	0.732	0.095	0.271	0.103	0.333	0.341	0.086	0.175	0.356	0.201	0.686	0.709	0.327	0.641	0.334	0.365	0.737	0.213	**0.005**

The numbers of intraspecific distances are shown in boldface for clarity

^a^ Interspecific distances of *An. hyrcanus* & *An. pseudopictus*, *An. hyrcanus* & *An. hyrcanus* sp_IR_, *An. hyrcanus* sp_IR_ & *An. pseudopictus*, *An. lesteri* & *An. paraliae*, *An. kleini* & *An. engarensis*

^b^ The highest intraspecific distance and the lowest interspecific distance

*Abbreviations*: *n* no. of sequences, *na* not applicable, *arg*. *An. argyropus, bel*. *An. belenrae*, *cra*. *An. crawfordi, eng*. *An. engarensis, hyr*. *An. hyrcanus, hyr*._*sp*_*IR*_ *An. hyrcanus* sp_IR,_ *kle*. *An. kleini*, *kwe*. *An. kweiyangensis, les*. *An. lesteri, lia*. *An. liangshanensis, nig*. *An. nigerrimus*, *nit*. *An. nitidus, par*. *An. paraliae, ped*. *An. peditaeniatus, pse*. *An. pseudopictus*, *pul*. *An. pullus, pur*. *An. pursati, siner*. *An. sineroides*, *sin*. *An. sinensis*

### Genetic diversity indices and neutrality tests

Additional file 4: Figure S2 displays the haplotype frequencies in Hyrcanus Group members. Sequence numbers for each species, polymorphic sites, haplotype numbers, haplotype diversity, and the Fu’s *Fs* and Tajima’s *D* results are given in Table 3. The presence of high haplotype diversity, combined with a positive neutrality test value, evidences balancing selection or a decrease in population size (e.g. *An. crawfordi*). Conversely, a relatively low haplotype diversity with a negative neutrality test value indicates that the population has experienced an expansion after a bottleneck, probably caused by purifying selection or a selective sweep due to genetic hitchhiking (e.g. *An. peditaeniatus*).

**Table 3:** Genetic diversity indices and neutrality tests (Fu’s *Fs* and Tajima’s *D*) of the ITS2 gene in 17 Hyrcanus Group members

Species	*n*	S	Pi	h	Hd	Fu’s *Fs*	Tajima’s *D*
*An. argyropus*	18	4	0.00289	4	0.477	0.584	0.49002
*An. nigerrimus*	26	2	0.00195	2	0.492	3.173	1.88188
*An. nitidus*	43	3	0.00222	3	0.638	2.118	1.12137
*An. pursati*	22	–	–	1	–	–	–
*An. pseudopictus*	30	–	–	1	–	–	–
*An. hyrcanus*	48	9	0.00140	5	0.268	-1.517	-1.98082*
*An. hyrcanus*_sp_IR_	7	5	0.00497	3	0.524	1.508	0.36328
*An. pullus*	23	4	0.00304	6	0.775	-1.211	0.60421
*An. lesteri*	18	42	0.02042	6	0.490	4.914*	-1.33520
*An. paraliae*	25	–	–	1	–	–	–
*An. crawfordi*	53	12	0.01115	4	0.491	9.501**	2.50567*
*An. liangshanensis*	4	1	0.00114	2	0.500	0.172	-0.61237
*An. sineroides*	4	1	0.00120	2	0.500	0.172	-0.61237
*An. sinensis*	41	5	0.00340	4	0.450	1.599	0.43762
*An. belenrae*	5	–	–	1	–	–	–
*An. kleini*	9	–	–	1	–	–	–
*An. peditaeniatus*	83	13	0.00094	9	0.185	-8.629**	-2.36082**

The significance of Fu’s *Fs* and Tajima’s *D* values is indicated by asterisks (*P < 0.05, **P < 0.01)

Species represented by < 3 specimens were excluded from the analyses

*Abbreviations*: *n* number of sequences, *S* number of polymorphic sites, *pi* nucleotide diversity, *h* number of haplotypes, *Hd* haplotype diversity

### Phylogenetic analysis

The Hyrcanus Group is monophyletic (Fig. 1). All lineages, including individuals representing the same species, were supported by high bootstrap values, except for *An. pseudopictus*, *An. pseudopictus* and *An. hyrcanus*, which exhibited barcode congruence with an extremely low interspecific distance (0.001). Low genetic divergences were also observed between *An. hyrcanus* and *An. hyrcanus* sp_IR_ (0.021), *An. lesteri* and *An. paraliae* (0.048), and *An. kleini* and *An. engarensis* (0.072). Each of these species was placed in an independent branch and was homologous to its closest taxon in the tree, suggesting that they might be candidate species or have diverged recently.

Although the ITS2-based phylogenetic tree was concordant with traditional morphological taxonomy in terms of species recognition, its subgroup arrangement did not match that obtained with morphology-based grouping. Following the NJ-K2P analysis, two main clusters were identified within the Hyrcanus Group. One consisted of the Nigerrimus Subgroup plus *An. argyropus* (Swellengrebel, 1914), and the other contained the Lesteri Subgroup and the unassigned species *An. hyrcanus*, *An. hyrcanus* sp_IR_, *An. pseudopictus*, *An. sinensis*, *An. belenrae*, *An. kleini*, *An. engarensis*, *An. liangshanensis*, *An. kweiyangensis* Yao & Wu, 1944 and *An. pullus*.

### Multiplex PCR assay

**Fig. 3:**
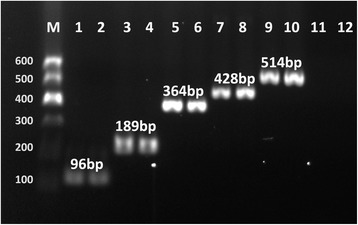
Polymerase chain reaction (PCR) products of a multiplex PCR assay. Lane M: PCR marker; Lanes 1 and 2: *An. peditaeniatus*; Lanes 3 and 4: *An. hyrcanus*; Lanes 5 and 6: *An. lesteri*; Lanes 7 and 8: *An. pullus*; Lanes 9 and 10: *An. sinensis*; Lanes 11 and 12: negative controls

Based on species-specific differences, six primers were combined in a multiplex PCR mixture for the simultaneous amplification of five Hyrcanus Group members from China (Fig. 3). The lengths of the amplified species-specific PCR products were approximately 96 bp for *An. peditaeniatus*, 189 bp for *An. hyrcanus*, 364 bp for *An. lesteri*, 428 bp for *An. pullus* and 514 bp for *An. sinensis*. To validate the assay, 92 specimens were tested, including 24 *An. peditaeniatus*, 10 *An. hyrcanus*, 3 *An. lesteri*, 11 *An. pullus* and 44 *An. sinensis*. All specimens that were confirmed by molecular identification yielded a specific band for each species, as expected. The specificity of the multiplex PCR was evaluated for its cross-reactivity with closely related organisms. No amplified product was observed with DNA of other species, even on repeated tests (Additional file 5: Figure S3). The sensitivity tests revealed that the minimum detection limit of mosquito DNA by the multiplex PCR system was 10^−4^ ng/μl (Additional file 6: Figure S4).

## Discussion

### Subdivision of the Hyrcanus group

Morphological features separate the Hyrcanus Group into three subgroups [52, 53]. *Anopheles nigerrimus*, *An. nitidus* Harrison, Scanlon & Reid, 1973, *An. pursati* and *An. pseudosinensis* Baisas, 1935 were placed in the Nigerrimus Subgroup, and *An. lesteri*, *An. paraliae*, *An. peditaeniatus*, *An. crawfordi* and *An. vietnamensis* Nguyen, Tran & Nguyen, 1993 were assigned to the Lesteri Subgroup. The remaining species were unassigned. Unfortunately, DNA barcoding has not been conducted for every Hyrcanus Group member. Until now, no ITS2 sequence data were known for the following seven species: *An. pseudosinensis*, *An. vietnamensis*, *An. chodukini* Martini, 1929, *An. hailarensis* Xu & Luo, 1998, *An. heiheensis* Ma, 1981, *An. pursati* and *An. nimpe* Nguyen, Tran & Harbach, 2000. The present study included all the ITS2 sequences available for Hyrcanus Group members deposited in GenBank, including 461 individuals from 19 species. The NJ tree obtained supports the monophyly of the Hyrcanus Group; however, the subgroup arrangement did not match that based on morphological characteristics. We could recognize two main subgroups, one consisting of *An. argyropus*, *An. nigerrimus*, *An. pursati* and *An. nitidus*, and the other containing *An. peditaeniatus*, *An. pullus*, *An. hyrcanus* sp_IR_, *An. hyrcanus*, *An. pseudopictus*, *An. kleini*, *An. engarensis*, *An. belenrae*, *An. sinensis*, *An. sineroides*, *An. kweiyangensis*, *An. liangshanensis*, *An. crawfordi*, *An. lesteri* and *An. paraliae*. This arrangement suggested that the morphology-based Lesteri Subgroup is not monophyletic. *Anopheles lesteri*, *An. paraliae* and *An. crawfordi* formed a single clade, but *An. peditaeniatus* was far from these species in the NJ tree. In addition, the ITS2 sequences of *An. hyrcanus* and *An. pseudopictus* were almost identical, with an extremely low genetic distance (0.001). As in the results based on the *cox*1 gene [22], the interspecific was only 0.008, which is far below the maximum intraspecific distance, i.e. 0.017. *Anopheles pseudopictus* individuals were embedded in the *An. hyrcanus* lineage, in both ITS2 and *cox*1 trees [22], in agreement with previous findings comparing morphological characters and molecular markers between the two species and morphologically-intermediate forms [4]. Thus, *An. hyrcanus* and *An. pseudopictus* are most likely the same species, although further studies including crossing experiments are needed to confirm this hypothesis.

Results of the present and previous studies on genetic relationships among Hyrcanus Group members do not entirely coincide. In previous studies [11, 12, 16, 25, 26], both the number of species and the number of sequences used were far lower than those used in the present study. In addition, some species have been confirmed as synonymous within the Hyrcanus Group [1, 2]. Because previous studies did not include an appropriate number of species, particularly of the Nigerrimus Subgroup, it has been difficult to detect subgroups in the phylogenetic tree. Hwang [12] classified the Hyrcanus Group into four subgroups, based on ITS2 data; however, in his maximum likelihood tree topology, the four subgroups were not on the same phylogenetic level. It should be noted that the above study [12], and two related studies [11, 16], used the same *An. crawfordi* ITS2 sequence (AF261949) to reconstruct the molecular phylogeny of the Hyrcanus Group. However, in our analysis, this sequence belongs to an originally misidentified specimen. In these three studies [11, 12, 16], the tree topologies suggested that *An. crawfordi* is closest to *An. peditaeniatus*, in contrast to the results of the present study and those of another recent study [26], in which *An. crawfordi* is distant from *An. peditaeniatus* but close to *An. lesteri*.

### Deep ITS2 intraspecific divergence in *An. lesteri* and *An. crawfordi*

As shown in Table 2, deep intraspecific differences were detected in *An. lesteri* and *An. crawfordi* (0.0142 and 0.0143, respectively), while all other species analysed presented intraspecific divergences below 0.005. Deep ITS2 divergences within species might be due to hidden diversity. Hwang [12] stated that *An. lesteri* could be divided into at least three types: A (the dominant type), B (AJ620899 and AJ620902) and C (AJ620900 and AJ620901). He also suggested that the Philippines type (AY375469) of *An. lesteri* proposed by Rueda et al. [54] should belong to type A. However, in the current study nucleotide substitution and insertions of AY375469 were not as frequent close to type A as to types B and C sequences, although this accession still represented a separate branch from *An. lesteri* type A in the tree. Therefore, the Philippines type is probably an independent *An. lesteri* type. For *An. crawfordi*, sequences could generally be differentiated into Cambodia and Thailand types, with support values of 98 and 79%, respectively. This genetic divergence probably resulted from geographical isolation [55].

### Phylogenetic reconstructions using *cox*1 and ITS2

**Fig. 4:**
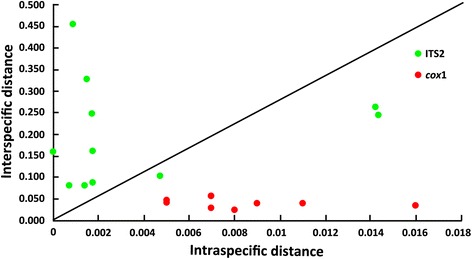
ITS2 and *cox*1 sequence divergences in the Hyrcanus Group. The minimum interspecific (intergroup) divergence is plotted against the maximum intraspecific divergence. ITS2 has more dots above the diagonal line in the top left-hand corner than *cox*1, indicating low intraspecific and high interspecific divergence

Based on the results of the present study and our previous study using *cox*1 [22], we compared the effectiveness of the nuclear ITS2 and mitochondrial *cox*1 as markers for Hyrcanus Group members. We found that the *cox*1 barcoding gap was 0.016–0.026, whereas that of ITS2 was 0.014–0.081. The ITS2 sequence divergences were, on average, almost 160 times higher among groups of species than within a species, whereas the average *cox*1 divergence between congroup species was only eight times higher than that within species. An effective DNA marker should have a small intraspecific distance and a large interspecific distance [56]. In Fig. 4, each dot represents a species, with intraspecific distance on the x-axis and interspecific distance on the y-axis. It is obvious that there are more ITS2 than *cox*1 dots close to the top left-hand corner of the graph. The main disadvantage of using *cox*1 in phylogenetic studies of the Hyrcanus Group is that it cannot differentiate between the closely related: *An. lesteri* and *An. paraliae*; and *An. sinensis*, *An. belenrae* and *An. kleini* [22]. In the ITS2 tree, each of the above species belonged to an independent lineage, which form monoclades with their sister species. In addition, two other pairs of sibling species, *An. hyrcanus* and *An. hyrcanus* sp_IR_, and *An. kleini* and *An. engarensis*, were also differentiated by ITS2. Currently, there are no *cox*1 records of *An. hyrcanus* sp_IR_ or *An. engarensis* in GenBank. Thus, rDNA ITS2 seems more reliable than mtDNA *cox*1 for resolving evolutionary issues of the Hyrcanus Group, including recently diverged taxa, such as cryptic species of mosquitoes. Although *cox*1 may be useful in barcoding information, and particularly for inferring the possibility of ancient hybridization, in mosquito molecular phylogeny, nuclear ITS2 can establish species boundaries in cases that cannot be resolved by mitochondrial *cox*1. In fact, ITS2 sequences have been most frequently used in species identification and phylogenetic reconstruction of the Hyrcanus Group [11, 12, 16, 25, 26]. In the animal kingdom, the evolutionary rate of the mitochondrial genome is about 5–10 times faster than that of the nuclear genome, making *cox*1 potentially more useful than ITS2 for correctly identifying recently diverged species [57, 58]. However, the opposite seems to occur within the Hyrcanus Group, which might be explained by the male-biased dispersal [59]. In the field, females usually mate only once, and store sufficient sperms to fertilize all eggs they produce in their lifetime, whereas males mate repeatedly. Consequently, male mosquitoes occupy a dominant position during population expansions. Because mtDNA is typically maternally inherited, any hybrid or offspring would only have the maternal species’ mtDNA. Therefore, hybridization can result in shared or very similar sequences in the mitochondrial genome. The divergent mtDNA of *An. belenrae*, *An. kleini* and *An. paraliae* suggests that the mitochondrial genomes of incipient sibling species have been sympatrically replaced by those of *An. sinensis* and *An. lesteri* over wide areas. In the Hyrcanus Group, nuclear markers are less introgressed, and hence more diagnostic, than mtDNA markers.

## Conclusions

This study used ITS2 sequences of Hyrcanus Group members distributed worldwide, providing a systematic basis for future studies on malaria transmission and its relationship with the evolution of *Plasmodium* spp. We found that the topology of the Hyrcanus Group ITS2 tree was generally consistent with the morphology-based taxonomy in terms of species classification, but not in terms of subgroup divisions. Nuclear and mitochondrial gene data were generally consistent in subgroup classification; however, *cox*1 failed to elucidate the phylogenetic status of the incipient sibling species *An. belenrae*, *An. kleini* and *An. paraliae*, suggesting that *cox*1 might be unable to resolve the molecular phylogeny of the Hyrcanus Group, unlike ITS2. ITS2 is a reliable tool for the study of phylogenetic relationships between closely related mosquito taxa, and *cox*1 may be a useful supplement to barcoding information, particularly for inferring interspecific hybridization. Both *cox*1 and ITS2 results suggested that *An. pseudopictus* and *An. hyrcanus* might be the same species. Two new ITS2 lineages, namely accessions AF261949 and KC769647, were uncovered, and require further sampling and detailed morphological, genetic, and ecological studies before resolving their true taxonomic status. In addition, unambiguous ITS2 sequence differences among Hyrcanus Group members facilitated the design of species-specific primers for identifying five species within this group that are the most frequently implicated in disease transmission in China. Thus, this method can identify members of the Hyrcanus Group in a simple, fast, and reliable manner during malaria-vector surveillance procedures.

## Additional files

Additional file 1: Table S1. List of the *Anopheles hyrcanus* group ITS2 sequences deposited in GenBank and obtained from this study, with GenBank accession numbers, geographical location, and corresponding authors. (XLSX 50 kb)

Additional file 2: Table S2. List of the specimens used in the multiplex PCR validation. (PDF 303 kb)

Additional file 3: Figure S1. Intra- and interspecific ITS2 divergences among the 19 Hyrcanus Group members as determined by Nei’s distance. (TIFF 779 kb)

Additional file 4: Figure S2. ITS2 haplotype frequencies in the 19 Hyrcanus Group members. (TIFF 145 kb)

Additional file 5: Figure S3. Partial results of the specificity test of the Multiplex PCR. (PDF 413 kb)

Additional file 6: Figure S4. Partial results of the sensitivity test of the Multiplex PCR for the five Hyrcanus Group members. (PDF 475 kb)

## Abbreviations

China CDC: Chinese Center for Disease Control and Prevention
*cox*1: Cytochrome *c* oxidase subunit 1 gene
ITS2: Internal transcribed spacer 2
K2P: Kimura’s 2-parameter
NIPD: National Institute of Parasitic Diseases
