# Supplementary material for: Molecular phylogeny of Anopheles hyrcanus group members based on ITS2 rDNA
Source: Parasit Vectors. 2017 Sep 7;10:417. doi: 10.1186/s13071-017-2351-x (PMC5590201; doi:10.1186/s13071-017-2351-x)
Supplement: Supplementary file 2 — List of the specimens used in the multiplex PCR validation. (PDF 303 kb) [file 13071_2017_2351_MOESM2_ESM.pdf]

Table S2. List of the specimens used in the multiplex PCR validation

Species	Number of specimens	GenBank ID
An. peditaeniatus	24	KU312204–KU312209; MF535198; MF498513–MF498529
An. hyrcanus	10	KU312217–KU312220; MF535199–MF535204
An. lesteri	3	KU312221; KU682193; MF535205
An. pullus	11	KU312210–KU312216; MF535206–MF535209
An. sinensis	44	KU312198–KU312203; MF535210–MF535218; MF498530–MF498558
An. jeyporiensis	3	MF535228–MF535230
An. minimus	4	MF535224–MF535227
An. harrisoni	2	MF535222–MF535223
An. maculatus	3	MF535219–MF535221
An. aconitus	3	MF535231–MF535233
An. splendidus	3	MF535234–MF535236
An. dravidicus	3	MF535237–MF535239
