# Supplementary figures and images for: Molecular phylogeny of Anopheles hyrcanus group members based on ITS2 rDNA
Source: Parasit Vectors. 2017 Sep 7;10:417. doi: 10.1186/s13071-017-2351-x (PMC5590201; doi:10.1186/s13071-017-2351-x)

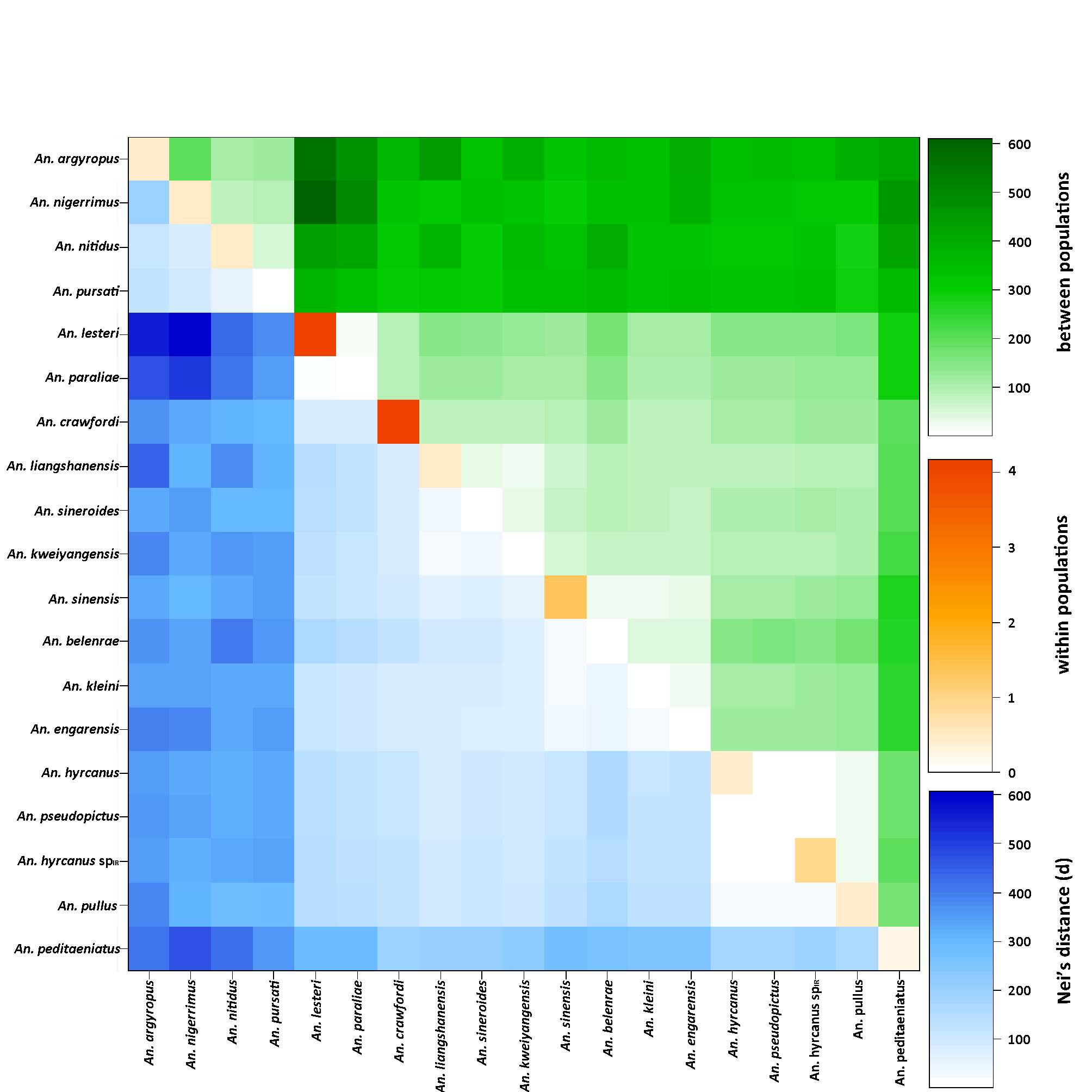

Supplement: Supplementary file 3 — Intra- and interspecific ITS2 divergences among the 19 Hyrcanus Group members as determined by Nei’s distance. (TIFF 779 kb) [file 13071_2017_2351_MOESM3_ESM.tif]

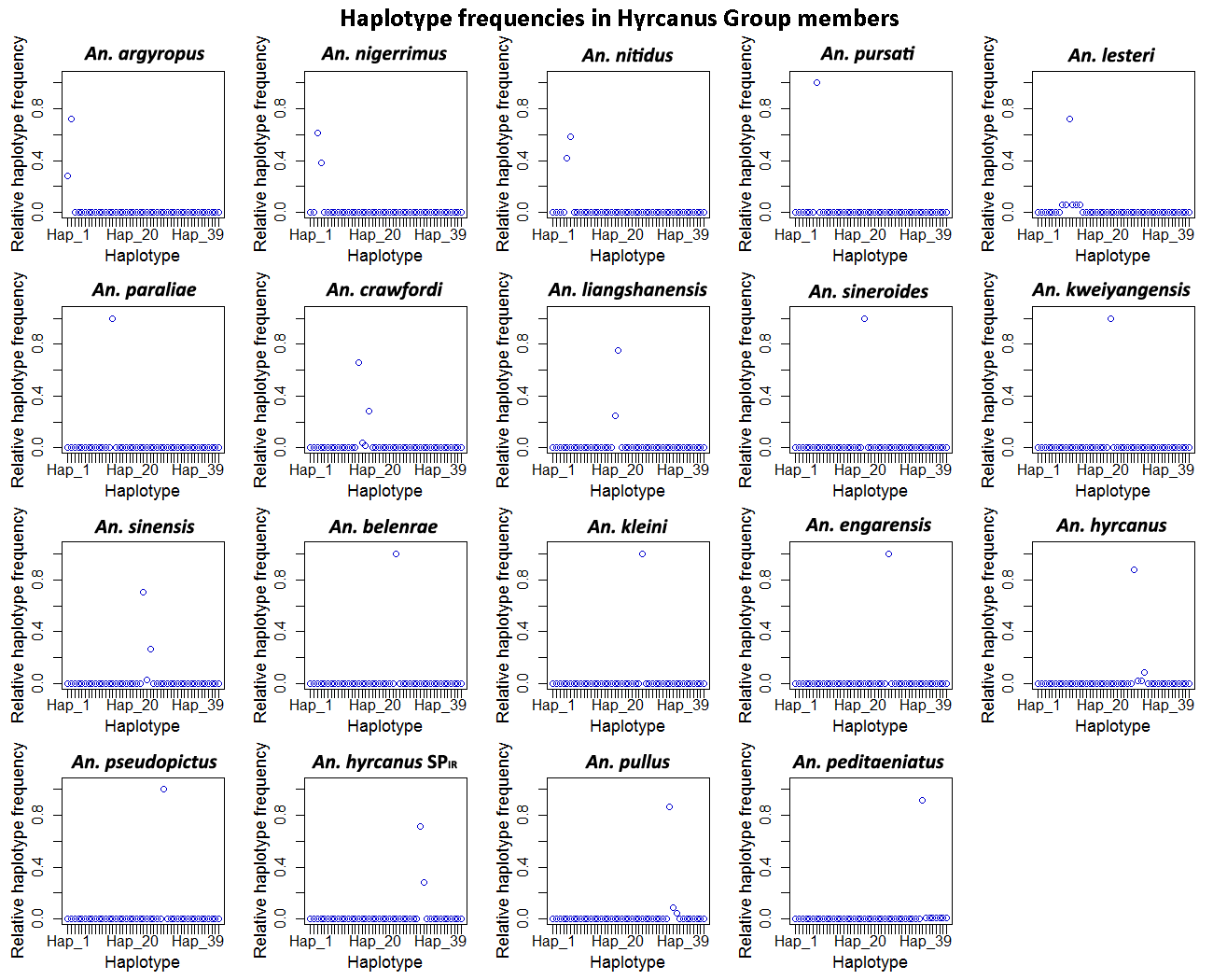

Supplement: Supplementary file 4 — ITS2 haplotype frequencies in the 19 Hyrcanus Group members. (TIFF 145 kb) [file 13071_2017_2351_MOESM4_ESM.tif]
