# Supplementary material for: Molecular phylogeny of Anopheles hyrcanus group members based on ITS2 rDNA
Source: Parasit Vectors. 2017 Sep 7;10:417. doi: 10.1186/s13071-017-2351-x (PMC5590201; doi:10.1186/s13071-017-2351-x)
Supplement: Supplementary file 5 — Partial results of the specificity test of the Multiplex PCR. (PDF 413 kb) [file 13071_2017_2351_MOESM5_ESM.pdf]

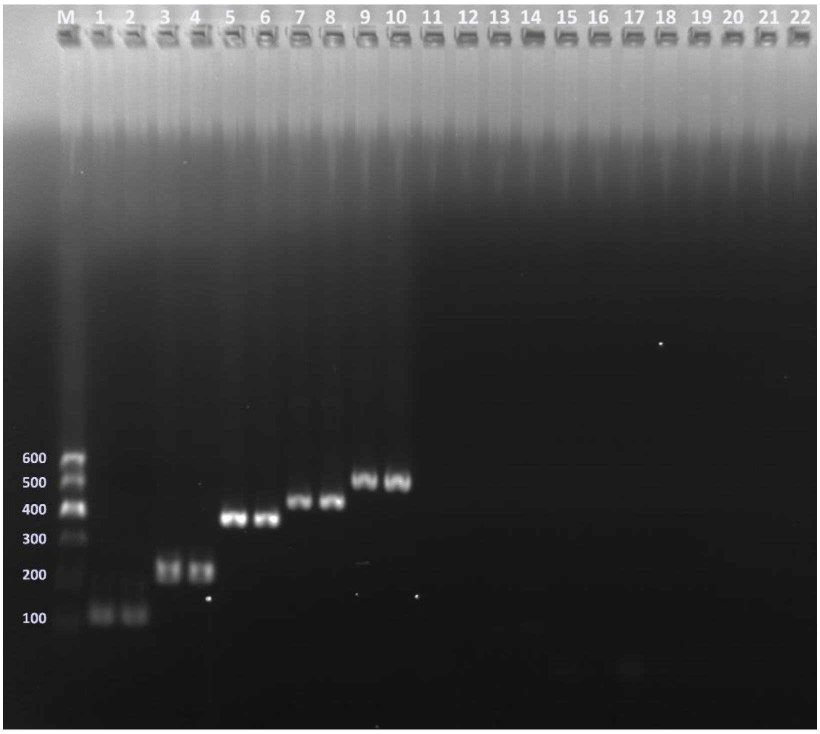

Figure S3 Partial results of the specificity test of the Multiplex PCR

M: DNA marker; Lanes 1 and 2: *An. peditaeniatus*; Lanes 3 and 4: *An. hyrcanus*; Lanes 5 and 6: *An. lesteri*; Lanes 7 and 8: *An. pullus*; Lanes 9 and 10: *An. sinensis*; Lanes 11 and 12: *An. liangshanensis*; Lanes 13 and 14: *An. minimus*; Lanes 15 and 16: *An. dirus*; Lanes 17 and 18: *An. harrisoni*; Lanes 19 and 20: *An. splendidus*; Lanes 21 and 22: negative control.
