# Supplementary material for: Molecular phylogeny of Anopheles hyrcanus group members based on ITS2 rDNA
Source: Parasit Vectors. 2017 Sep 7;10:417. doi: 10.1186/s13071-017-2351-x (PMC5590201; doi:10.1186/s13071-017-2351-x)
Supplement: Supplementary file 6 — Partial results of the sensitivity test of the Multiplex PCR for the five Hyrcanus Group members. (PDF 475 kb) [file 13071_2017_2351_MOESM6_ESM.pdf]

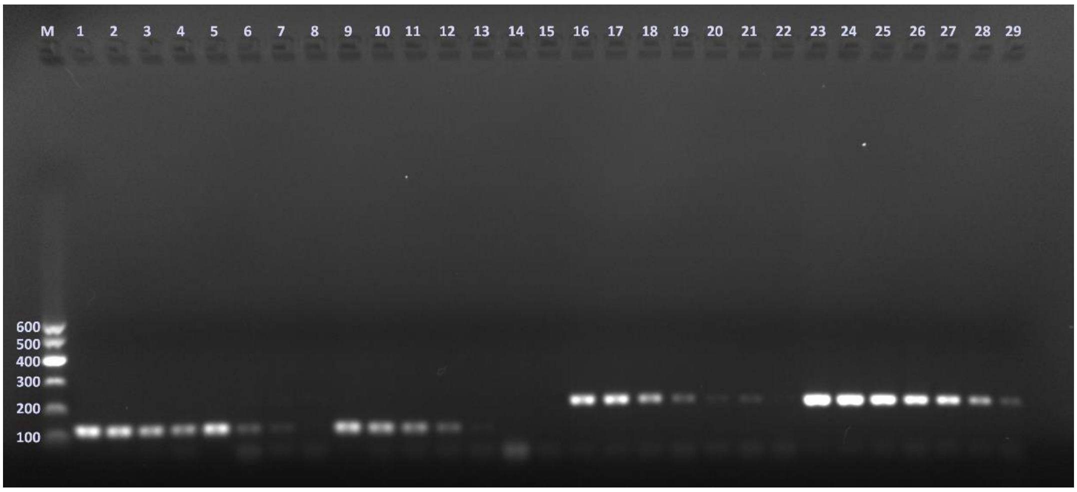

Figure S4A

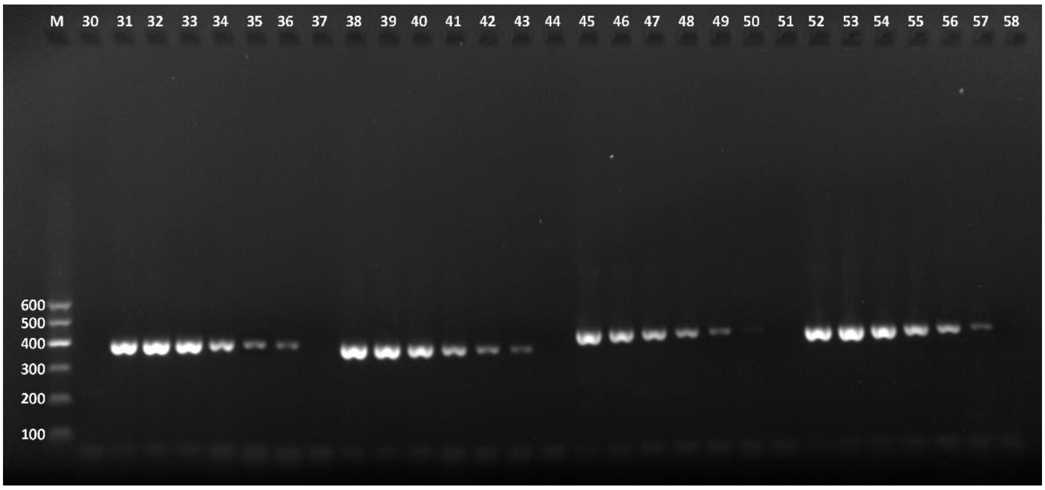

Figure S4B

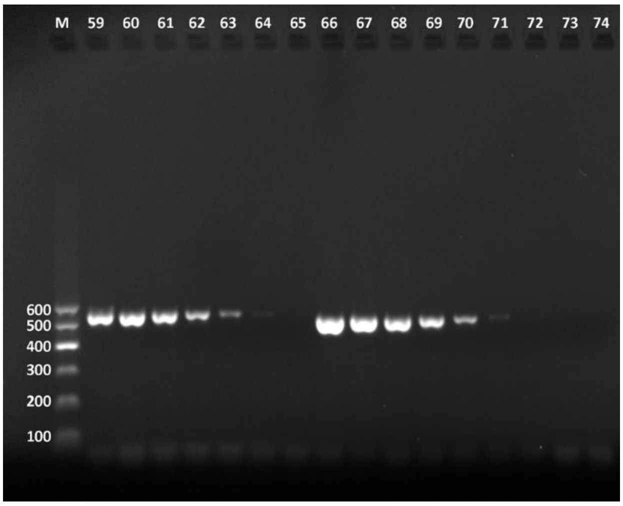

Figure S4C

Figure S4 Partial results of the sensitivity test of the Multiplex PCR for the five Hyrcanus Group members.

M: DNA marker; Lanes 1-8: concentration of *An. peditaeniatus* template diluting from 10 ng/µL

to 1×10^{-6} ng/μL in turn; Lanes 9-15: concentration of *An. peditaeniatus* template diluting from 10 ng/μL to 1×10^{-5} ng/μL in turn; Lanes 16-22: concentration of *An. hyrcanus* template diluting from 10 ng/μL to 1×10^{-5} ng/μL in turn; Lanes 23-30: concentration of *An. hyrcanus* template diluting from 10 ng/μL to 1×10^{-6} ng/μL in turn; Lanes 31-37: concentration of *An. lesteri* template diluting from 10 ng/μL to 1×10^{-5} ng/μL in turn; Lanes 38-44: concentration of *An. lesteri* template diluting from 10 ng/μL to 1×10^{-5} ng/μL in turn; Lanes 45-51: concentration of *An. pullus* template diluting from 10 ng/μL to 1×10^{-5} ng/μL in turn; Lanes 52-58: concentration of *An. pullus* template diluting from 10 ng/μL to 1×10^{-5} ng/μL in turn; Lanes 59-65: concentration of *An. sinensis* template diluting from 10 ng/μL to 1×10^{-5} ng/μL in turn; Lanes 66-72: concentration of *An. sinensis* template diluting from 10 ng/μL to 1×10^{-5} ng/μL in turn; Lanes 73 and 74: negative control.
